# Frequency Split Elimination Method for a Solid-State Vibratory Angular Rate Gyro with an Imperfect Axisymmetric-Shell Resonator

**DOI:** 10.3390/s150203204

**Published:** 2015-02-02

**Authors:** Zhen Lin, Mengyin Fu, Zhihong Deng, Ning Liu, Hong Liu

**Affiliations:** School of Automation, Beijing Institute of Technology, Beijing 100081, China; E-Mails: fumy@bit.edu.cn (M.F.); dzh_deng@bit.edu.cn (Z.D.); liuning1898@qq.com (N.L.); 13581903098@126.com (H.L.)

**Keywords:** vibratory gyro, axisymmetric shell, frequency split, vibration mode

## Abstract

The resonator of a solid-state vibratory gyro is responsible for sensing angular motion. Frequency splitting of an axisymmetric-shell resonator is a common problem caused by manufacturing defects. The defect causes a frequency difference between two working modes which consist of two nodes and two antinodes. The difference leads to the loss of gyroscopic effect, and thus the resonator cannot sense angular motion. In this paper, the resonator based on an axisymmetric multi-curved surface shell structure is investigated and an approach to eliminate frequency splits is proposed. Since axisymmetric multi-curved surface shell resonators are too complex to be modeled, this paper proposes a simplified model by focusing on a common property of the axisymmetric shell. The resonator with stochastic imperfections is made equivalent to a perfect shell with an imperfect mass point. Rayleigh's energy method is used in the theoretical analysis. Finite element modeling is used to demonstrate the effectiveness of the elimination approach. In real cases, a resonator's frequency split is eliminated by the proposed approach. In this paper, errors in the theoretical analysis are discussed and steps to be taken when the deviation between assumptions and the real situation is large are figured out. The resonator has good performance after processing. The elimination approach can be applied to any kind of solid-state vibratory gyro resonators with an axisymmetric shell structure.

## 1. Introduction

Solid-state wave gyroscopes arose as a new type of gyro at the end of 20th century [1]. Their basic principle is the Coriolis effect [2]. This kind of gyroscope can be used to measure the angular velocity of a rotating body by testing the inertia effect of standing waves [3]. In contrast to traditional gyros, the new gyros are simple, all-solid state, highly reliable [4], and low cost. In addition, they can resist high impacts.

The resonator is a key part of the vibratory gyro. There are various of structures such as beam [5], tuning fork and axisymmetric structures [6]. Axisymmetric structures includes hemispherical shell [7,8], cylindrical shell [9,10] and ring structures [11]. At present, the Hemispherical Resonator Gyro (HRG) is the Coriolis vibratory gyro with the highest accuracy and it is used in many mature products. In 1979, the Delco Electronics Corporation produced the first HRG. HRGs were first used in spatial inertial reference units in 1996. After 14 years of usage, the gyro had worked over 12-million operating gyro hours in total with an 100% mission success rate in space [12]. This indicates its high reliability and long lifespan. In this regard, the vibratory gyro with axisymmetric shell is very promising.

**Figure 1.**
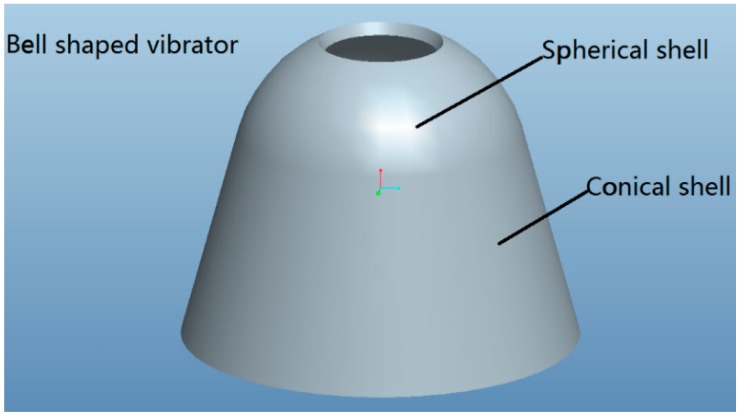
Axisymmetric multi-curved surface shell structure.

In the study of vibratory gyros with axisymmetric-shell resonators, Liu Ning presented an angular rate vibratory gyro called the bell-shaped vibratory gyro [13]. This vibratory gyro was inspired by a Chinese traditional bell based on an axisymmetric multi-curved surface shell structure. One such structure is shown in Figure 1. A bell-shaped vibratory gyro's impact resistance is up to 10,000 g. The performance of this gyro's impact resistance is much better than that of any other vibratory gyro, so that it can be widely used in weapon systems.

**Figure 2.**
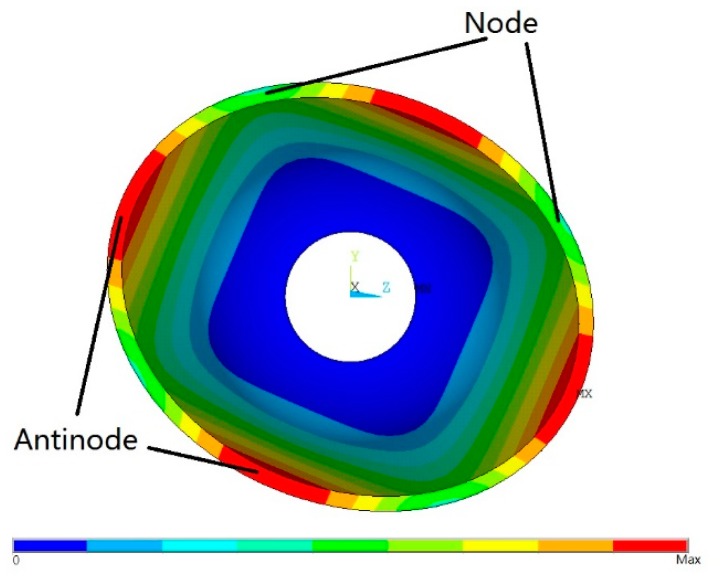
A bottom-up view of a resonator's working mode (different colors represent different amplitudes).

The working mode of a bell-shaped resonator is shown in Figure 2. From the perspective of the resonator's bottom, it has four antinodes. The axisymmetric shell has two sets of four-antinode modes. They are orthogonal to the structure [14] and they also have the same natural frequency. However, the imperfections caused by manufacturing defects will bring about frequency splits, *i.e.*, the two sets of four-antinode modes' natural frequencies are not equal. When a resonator has a frequency split, the vibration shape can also be influenced by the imperfections besides rotation and the Coriolis effect, and the gyroscopic effect will be lost. As a result, the frequency split must be eliminated. Xi, X. *et al.* discussed the impacts of frequency splits on cylindrical resonators and studied the relationship between the frequency split and the vibration axis shift angle of a vibrating cylindrical resonator [15], but his research did not include the elimination of the frequency split. Tao, Y. *et al.* presented ways to eliminate a cupped-shell resonator's frequency split with experiments [16]. Fox showed the impact of adding or trimming the mass point on a perfect ring resonator by adopting the Rayleigh-Ritz method [17]. Later, Fox built a model that adds an equivalent imperfect mass point to a perfect ring and proposed a method to eliminate the frequency split [18]. The frequency split of an imperfect hemispherical shell is also studied with the same method in [19].

However, the published literatures all focus on simple structures like ring, hemisphere and cylinder shells. In this paper, we studied the frequency split of an axisymmetric multi-curved surface shell resonator. The shape of bell-shaped resonator is very complex and its middle surface cannot be described by a single linear function, which makes it difficult to analyze. The starting point of this research is not to use a complicated mathematical description, but rather to simplify it by summarizing the similarities of an axisymmetric shell resonator's vibration. The equivalent imperfect mass point concept is applied in this paper. The aim of this paper is to propose an elimination method that adds balance mass at a given position with a given mass that is proven theoretically. In the FEM simulation and Experimental sections, we discuss the errors of the theoretical analysis, reduce its limitations, and prove the effectiveness of the elimination method. The proposed frequency split elimination method is suitable for all imperfect axisymmetric shell resonators.

## 2. Resonator's Analysis of Vibratory Mechanics

**Figure 3.**
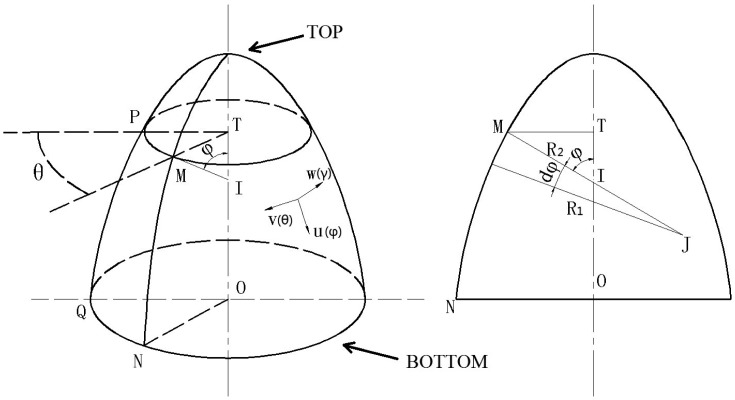
A resonator's curvilinear coordinates.

The imperfect axisymmetric multi-curved surface shell resonator can be equaled to a perfect axisymmetric multi-curved surface shell with one equivalent mass point at its bottom (see Figure 3). The equivalent model and real imperfect shell have the same natural frequency and mode shape. This equivalent model is based on the assumption that the imperfections are small enough so that the mode shape can be described by Equation (7). The theories of elasticity and vibration are applied.

### 2.1. Imperfect Resonator's Equivalent Model

An axisymmetric multi-curved surface shell resonator's curvilinear coordinates is shown in Figure 3, where φ stands for latitude, θ stands for longitude and γ stands for the dimension which is perpendicular to the φ-θ surface. *u*, *v*, and *w* represent the resonator's displacement in three directions. *R_1_* (MJ) and *R_2_* (MI) is main curvature radius at point M. *A* and *B* are supposed to be the Lame coefficients in φ and θ directions. Since the resonator is an axisymmetric shell, Equation (1) is obtained. *R_1_* and *R_2_* are functions that only depend on φ. We do not need to know the algebraic expressions of *R_1_* and *R_2_* in the analysis:

(1) $$A=R_{1}\left(\varphi \right),B=R_{2}\left(\varphi \right)\sin\left(\varphi \right)$$

The material of resonator is isotropic and uniform. It is assumed that the top of the resonator's displacement is 0. The Rayleigh-Ritz method is employed to calculate the resonator's natural frequency. The kinetic energy of vibrating prefect resonator is calculated by Equation (2):

(2) $$K_{0}=\frac{1}{2}\rho \int_{Q}^{T}\int_{0}^{2\pi }\int_{-h/2}^{h/2}\left({\dot{u}}^{2}+{\dot{v}}^{2}+{ẇ}^{2}\right)Bd\theta \cdot Ad\varphi d\gamma$$

Here ρ represents the density of the material. *Q* and *T* stand for the latitudes at the top and bottom of the resonator respectively. *h* stands for the thickness of the resonator. According to the shell theory hypothesis [20], the mode shape functions are expressed by Equation (3) [1,13]:

(3) $$\{\begin{array}{c} u\left(\varphi ,\theta ,\gamma ,\text{t}\right)=CU\left(\varphi \right)\cos\left(n\theta \right)\sin\left(\omega_{0}t\right)\\ \nu \left(\varphi ,\theta ,\gamma ,\text{t}\right)=CV\left(\varphi \right)\sin\left(n\theta \right)\sin\left(\omega_{0}\text{t}\right)\\ w\left(\varphi ,\theta ,\gamma ,\text{t}\right)=CW\left(\varphi \right)\cos\left(n\theta \right)\sin\left(\omega_{0}t\right) \end{array}$$

where *n* denotes the mode number. *n* is 2 when the resonator is working in four-antinode modes. ω*_0_* denotes the natural frequency of perfect shell and *C* denotes the magnitude of mode shape. *U*, *V* and *W* are the Rayleigh functions of the resonator. These functions also can be calculated numerically through a simple iteration process which is presented in [21]. The potential energy of a vibrating resonator is written as:

(4) $$P_{0}=\frac{1}{2}\int_{Q}^{T}\int_{0}^{2\pi }\int_{-h/2}^{h/2}\left(\sigma_{\varphi }e_{\varphi }+\sigma_{\theta }e_{\theta }+\sigma_{\varphi \theta }e_{\varphi \theta }\right)$$

where σ denotes stress and *e* denotes strain. The relationship between stress and strain can be found in [20]. According to the Rayleigh-Ritz method [22]:

(5) $$K_{0\max}=P_{0\max}$$

So:

(6) $$\omega_{0}^{2}=\frac{P_{0\max}}{\frac{1}{2}C^{2}\rho h\pi \int_{Q}^{T}\left(U^{2}+V^{2}+W^{2}\right)\sin\left(\varphi \right)R_{1}R_{2}d\varphi }$$

**Figure 4.**
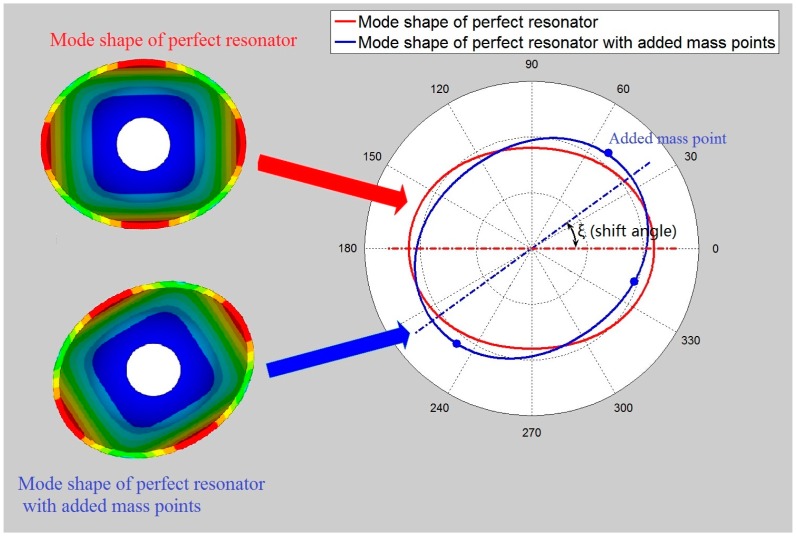
The contrast between the mode shape of a perfect resonator with and without added mass points.

Mass points are added to the bottom of the resonator. The mode shape of the resonator with added mass will change. It is assumed that the imperfections are so small that all other influences can be ignored, except for the shift angle ξ, as is shown in Figure 4. The coordinate of the *i*th added mass point is (*T*, θ*_i_*, 0) and the mass is *m_i_*, so the displacement of the added mass points on the resonator is shown below [19]:

(7) $$\{\begin{array}{c} u\left(\varphi ,\theta ,\gamma ,\text{t}\right)=CU\left(T\right)\cos\left[n(\theta_{i}-\xi )\right]\sin\left(\omega_{n}t\right)\\ v\left(\varphi ,\theta ,\gamma ,\text{t}\right)=CV\left(T\right)\sin\left[n(\theta_{i}-\xi )\right]\sin\left(\omega_{n}t\right)\\ w\left(\varphi ,\theta ,\gamma ,\text{t}\right)=CW\left(T\right)\cos\left[n(\theta_{i}-\xi )\right]\sin\left(\omega_{n}t\right) \end{array}$$

ω*_n_* denotes the natural frequency of the shell with added mass points. The total kinetic energy of resonator with mass points is:

(8) $$K_{T}=K_{0}+K_{m}$$

where *K_m_* denotes the kinetic energy of added mass point. The change of resonator's potential energy can be ignored after adding the mass points [19]. The natural frequency of shell with mass points is denoted by ω*_0_* in Equation (9):

(9) $$\omega_{n}^{2}=\omega_{0}^{2}{\left(1+\frac{\sum_{i}m_{i}\left\{U{\left(T\right)}^{2}\cos^{2}\left[n\left(\theta_{i}-\xi \right)\right]+V{\left(T\right)}^{2}\sin^{2}\left[n\left(\theta_{i}-\xi \right)\right]+W{\left(T\right)}^{2}\cos^{2}\left[n\left(\theta_{i}-\xi \right)\right]\right\}}{\rho \pi h\int_{Q}^{T}\left(U^{2}+V^{2}+W^{2}\right)\sin\left(\varphi \right)R_{1}R_{2}d\varphi }\right)}^{-1}$$

ω*_0_* is expressed by Equation (6). ξ is the stationary value of ω*_n_* [19], so:

(10) $$\frac{\partial \omega_{n}^{2}}{\partial \xi }=0$$

Substitute Equation (9) into Equation (10):

(11) $$\tan\left(2n\xi \right)=\frac{\sum_{i}m_{i}\sin\left(2n\theta_{i}\right)}{\sum_{i}m_{i}\cos\left(2n\theta_{i}\right)}$$

The shift angle ξ can be calculated by Equation (11).

Besides the discussion of how added mass points influence a perfect shell, now the paper will discuss how to make an imperfect shell equivalent to a perfect shell with one mass point. The equivalent mass point is attached at the bottom (φ = *T*) of the perfect shell. Suppose θ*_p_* is the position of equivalent imperfect mass point, and *m_p_* is the mass, thus shift angle ξ can be calculated by Equation (11):

(12) $$\{\begin{array}{l} \xi_{L}=\theta_{p}\\ \xi_{H}=\theta_{p}+\frac{\pi }{2n} \end{array}$$

Since two different shift angles are concluded from Equation (12), two different natural frequencies can be calculated by substituting Equation (12) into Equation (9). To obtain the obvious gyroscopic effect, the resonator of the gyro is designed to have a large amplitude in the γ direction [13]. This indicates that *W(T)^2^* > *V(T)^2^* and *U(T)^2^* > *V(T)^2^*, so:

(13) $$\{\begin{array}{c} \omega_{nH}^{2}=\omega_{0}^{2}{\left(1+\frac{m_{p}G_{H}}{S}\right)}^{-1}\\ \omega_{nL}^{2}=\omega_{0}^{2}{\left(1+\frac{m_{p}G_{L}}{S}\right)}^{-1} \end{array}$$

where:

(14) $$\{\begin{array}{l} S=\rho \pi h\int_{Q}^{T}\left(U^{2}+V^{2}+W^{2}\right)\sin\left(\varphi \right)R_{1}R_{2}d\varphi \\ G_{j}=U{\left(T\right)}^{2}\cos^{2}\left[n\left(\theta_{p}-\xi_{j}\right)\right]+V{\left(T\right)}^{2}\sin^{2}\left[n\left(\theta_{p}-\xi_{j}\right)\right]+W{\left(T\right)}^{2}\cos^{2}\left[n\left(\theta_{p}-\xi_{j}\right)\right],\left(j=L\text{or}H\right) \end{array}$$

ω*_nH_* is the higher natural frequency of the imperfect shell and ω*_nL_* is the lower one, so |ω*_nH_* − ω*_nL_*| is the frequency split. If we know all the parameters of the resonator and then measure ω*_nH_*, ω*_nL_*, and ξ*_L_* or ξ*_H_*, the mass (*m_p_*) and position (θ*_p_*) of the equivalent imperfect mass point is concluded:

(15) $$\{\begin{array}{l} m_{p}=\frac{S\left(\omega_{nH}^{2}-\omega_{nL}^{2}\right)}{\omega_{nL}^{2}G_{L}-\omega_{nH}^{2}G_{H}}\\ \theta_{p}=\xi_{L} \end{array}$$

### 2.2. The Approach to the Elimination of Frequency Split

Uniformly distributed balance mass points are added to the bottom of the imperfect resonator (φ = *T*) to eliminate frequency split. The mode number *n* is 2. Suppose that *m_i_* and θ*_i_* (*i* = 1, 2, 3…) denote the mass and position of balance mass points. Then θ*_i_* and *m_i_* can be expressed by Equation (16):

(16) $$\{\begin{array}{l} \theta_{i}=\frac{\left(i-1\right)*2\pi }{N}+\theta_{1}\left(i=1,2,3,\ldots ,N\right)\\ m_{i}=M/N \end{array}$$

**Figure 5.**
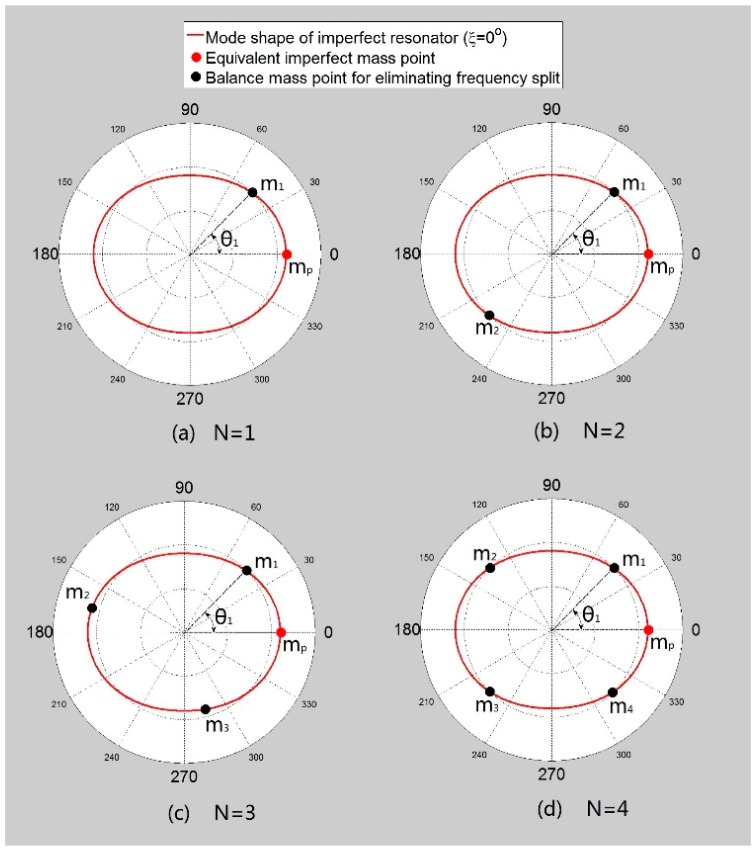
Distribution of equivalent imperfect mass point and balance mass points (*N* = 1, 2, 3, 4).

M is the total mass of balance mass points. N is the number of balance mass points (“N” and “*n*” are different parameters.). Assuming that the shift angle (ξ*_L_*) of the imperfect shell mode shape is 0 and the mass of the equivalent imperfect mass point is *m_p_*, the equivalent imperfect mass point's position θ*_p_* is 0, as shown in Figure 5. Firstly, we begin with adding one balance mass point (*i.e.*, *N* = 1). Figure 5a shows the mass point distribution.

Substituting the parameters into Equation (9):

(17) $$\{\begin{array}{c} \omega_{nL}^{2}=\omega_{0}^{2}{\left(1+\frac{m_{p}E+ME+ZF}{S}\right)}^{-1}\\ \omega_{nH}^{2}=\omega_{0}^{2}{\left(1+\frac{m_{p}E+ME-ZF}{S}\right)}^{-1} \end{array}$$

where:

(18) $$\{\begin{array}{c} E=\frac{1}{2}\left[U{\left(T\right)}^{2}+V{\left(T\right)}^{2}+W{\left(T\right)}^{2}\right]\\ F=\frac{1}{2}\left[U{\left(T\right)}^{2}-V{\left(T\right)}^{2}+W{\left(T\right)}^{2}\right]\\ Z=m_{p}\cos\left[4\left(\theta_{p}-\xi_{L}\right)\right]+M\cos\left[4\left(\theta_{1}-\xi_{L}\right)\right] \end{array}$$

**Figure 6.**
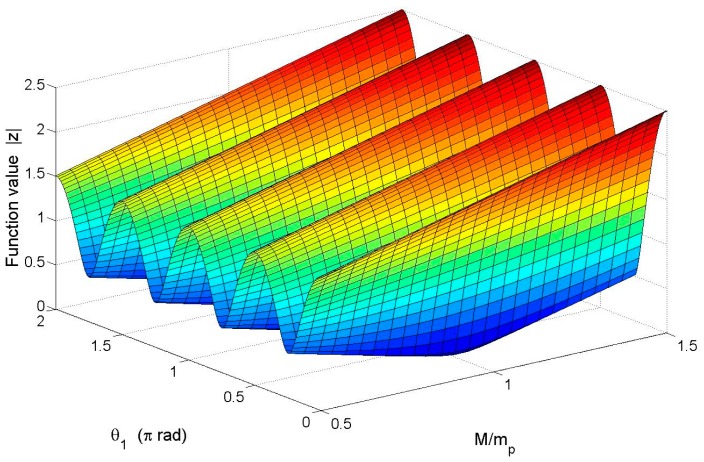
The relationship between |*Z*| and θ*_1_* as well as that between |*Z*| and *M*.

From Equation (17), we conclude that the absolute value of function *Z* plays a decisive role in the frequency split. The frequency split is getting smaller when the absolute value of *Z* is getting smaller. If *Z* = 0 then the frequency split will disappear. According to Equation (18) and Equation (11), Z can be calculated. Figure 6 respectively shows the relationship between the absolute value of *Z* and θ*_1_* as well as that between the absolute value of *Z* and total mass *M* (here *M* = *m_1_*).

θ*_1_* ranges from 0 rad to 2π rad and *M* ranges from 0.5·*m_p_* to 1.5·*m_p_* (see Figure 6). The figure shows that |*Z*| is varying wavily along θ*_1_* when *M* is a constant. |*Z*| reaches its minimum when θ*_1_* equals either 0.25π, 0.75π, 1.25π, or 1.75π. These four values of θ*_1_* can be the positions where balance mass points are added. When *M* is smaller than *m_p_*, the peak-to-peak value of |*Z*| is positively correlated to the value of *M*. When *M* is larger than *m_p_*, the peak-to-peak value of |*Z*| remains stable while the value of |*Z*| keeps moving upward. When *M* equals *m_p_* and θ*_1_* equals either 0.25π, 0.75π, 1.25π or 1.75π |*Z*| will be 0, and the frequency split disappears.

Secondly, if we add two balance mass points (*i.e.*, *N*= 2), the position of the balance mass points are θ*_1_*, θ*_2_* and the mass is *m_1_*, *m_2_*, where θ*_1_* = θ*_2_* + *π*. Figure 5b shows the distribution. Substituting those parameters into Equations (9) and (11), the conclusion is similar to the result of adding one balance mass point (here *M* = *m_1_* + *m_2_*). If *M* equals *m_p_* and θ*_1_* equals either 0.25π, 0.75π, 1.25π or 1.75π, the frequency split disappears.

Thirdly, if we add four balance mass points (*i.e.*, *N* = 4) as is shown in Figure 5d, the conclusion is also similar to adding one point (here *M* = *m_1_* + *m_2_* + *m_3_* + *m_4_*). Finally, we consider other values of N (*i.e.*, *N* = 3, 5, 6…). Figure 5c shows the distribution when *N* = 3. According to the symmetry of mass points' distribution and the Dirichlet formula, we conclude:

(19) $$\{\begin{array}{ll} \begin{array}{c} \sum_{i}m_{i}\sin\left(2n\theta_{i}\right)=0\\ \sum_{i}m_{i}\cos\left(2n\theta_{i}\right)=0 \end{array} & \left(\text{when}N\neq 1,2,4\right) \end{array}$$

Substituting Equation (19) into Equation (11), we conclude that ξ*_L_* = θ*_p_*. Then, substituting the conclusion into Equation (9):

(20) $$\{\begin{array}{c} \omega_{nL}^{2}=\omega_{0}^{2}{\left(1+\frac{m_{p}E+ME+m_{p}F}{S}\right)}^{-1}\\ \omega_{nH}^{2}=\omega_{0}^{2}{\left(1+\frac{m_{p}E+ME-m_{p}F}{S}\right)}^{-1} \end{array}$$

According to Equation (20), when *N* ≠ 1, 2 or 4, no matter what the values of *m_i_* and θ*_i_* are, the frequency split cannot be eliminated (since *m_i_* and θ*_i_* satisfy Equation (16)).

### 2.3. Theoretical Result and Discussion

The frequency split model is established by using the equivalent imperfect mass point model. Mass and position of an equivalent mass point are calculated by Equation (15). One thing to note is that all previous analyses are based on the hypothesis of a thin shell and Equation (7)'s assumption. The approach to eliminate the frequency split is concluded through theoretical analysis, which is to add one, two or four balance mass points (*N* = 1, 2, or 4) to the resonator to form a 0.25π-rad angle with the antinode of the lower-natural-frequency mode (there are four antinodes when *n* = 2) in the θ direction. The total mass of the balance mass point is equal to the mass of the equivalent imperfect mass point.

Although frequency splits can be eliminated when some kind of unevenly distributed balance mass points are added to the resonator, uniformly distributed balance mass points will not make the center of mass deviate from the symmetric axis of the resonator. Therefore, the uniformly distributed mass points can make the real mode shape closer to the assumption (Equation (7)) and the theoretical analysis closer to reality (this will be validated in Section 3). This paper only discusses the method to eliminate frequency splits by adding balance masses. The method of trimming (removing) masses can be concluded with the same analysis, but we will not discuss it any further in this article.

## 3. FEM Simulation of the Elimination Method

### 3.1. Simulation Conditions

**Figure 7.**
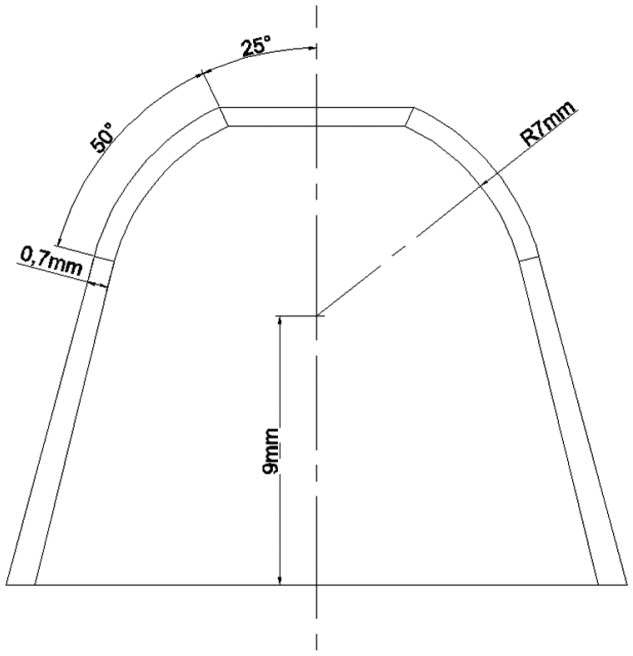
The size of resonator which is simulated.

The finite element method (FEM) is used to verify the conclusions mentioned above. The shape of resonator is shown in Figure 7. The FEM software ANSYS is applied. Element SHELL 63 is employed to simulate the perfect shell. Element MASS 21 is used to represent the added mass point. The material is Ni43CrT. It retains a stable modulus of elasticity when the temperature changes [3]. The properties of the material are shown in Table 1.

The elimination method drawn from above is based on Equation (7)'s assumption, so the gap between the actual mode shape of an imperfect shell and the mode shape assumed by Equation (7) may affect the validity of the elimination method. In the simulation, different gap levels are set and their influence on the elimination method is discussed. Frequency splits are created by adding calculated mass points to the perfect shell to make it imperfect. Different frequency split cases with different levels of mode shape gap should have the same equivalent mass point models in the simulation. In order to achieve an obvious simulation result, the mass of an equivalent imperfect mass point (*m_p_*) is 0.04 g, the frequency split is about 120 Hz and the shift angle ξ*_L_* is 0°.

It is well known that the accuracy of FEM results depends on the mesh size. Simulation results with different mesh sizes are shown in Table 2. It is obvious that the FEM induces a frequency split even in a perfect resonator model. Under the abovementioned simulation conditions, the perfect shell is simulated and the frequency split is about 0.2 Hz. This error can be ignored, compared to deliberately creating a frequency split (about 120 Hz). Table 2 also shows that the changes of the simulation results are slight when the latitude division is over 360 and longitude division is over 50, so the latitude division is set at 360 and the longitude division is set at 60 and the influence of mesh size can be ignored.

### 3.2. Simulations and Results

**Figure 8.**
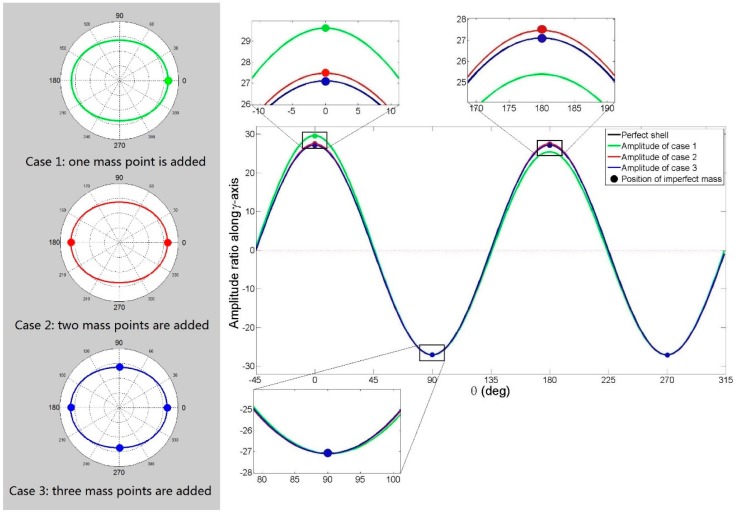
The mode shape of the perfect and imperfect resonator (the amplitude ratio is along the γ-axis at the resonators' bottom, negative amplitudes represent the phase difference of π rad).

*Frequency split case 1*: one imperfect mass point is added to the bottom of a perfect shell (mass is 0.04 g and position θ is 0°), as the tiny green dot shows in Figure 8; *Case 2*: two imperfect mass points are added (each has a mass of 0.02 g and positions of 0° and 180°), as the tiny red dots show in Figure 8; *Case 3*: four imperfect mass points are added (each has a mass of 0.01 g and a positions of 0°, 90°, 180° and 270°), as the tiny blue dots show in Figure 8. According to Equations (11) and (15), the mass and positions of the three frequency split cases' equivalent mass points all satisfy the simulation conditions (mass (*m_p_*) is 0.04 g, ξ*_L_* is 0°).

Mode shapes of the imperfect and perfect resonator are computed by FEM software. Figure 8 shows imperfect shells' and perfect shell's amplitude ratio along the γ-axis at the resonators' bottom. If imperfections are not small enough, we can see the gap between the imperfect shell's amplitude and the sine curve as expressed in the assumption (Equation (7)). The gap is enlarged in Figure 8. As a result of the uniformly distributed imperfect mass, frequency split Case 3 is the closest one to Equation (7)'s assumption (see the black line in Figure 8), while Case 1 shows the maximum gap with Equation (7)'s assumption among the three frequency split cases.

**Figure 9.**
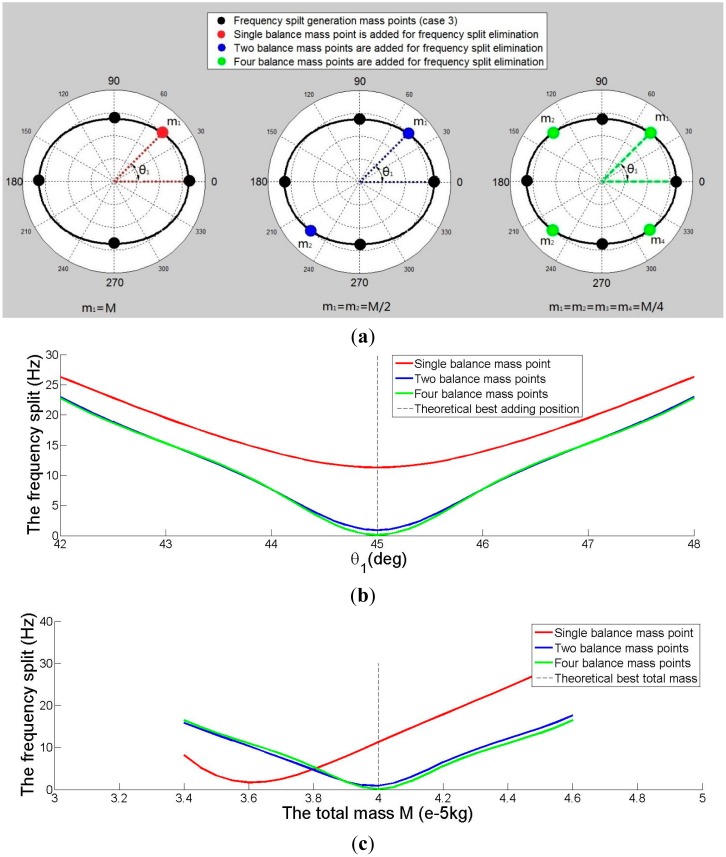
(**a**) The distribution of added mass points for eliminating frequency split Case 3; (**b**) The relationship between θ*_1_* and frequency split for eliminating frequency split Case 3 (*M* = 0.04 g); (**c**) The relationship between mass of balance points *M* and frequency split for eliminating frequency split Case 3 (θ*_1_* = π/4).

The elimination method is first applied to eliminate the frequency split of imperfect shell Case 3. The results are simulated by FEM software. According to the elimination methods, one balance mass point with a mass of *M* and with a position of θ*_1_*, or two balance mass points with masses of *M*/2 and with positions θ*_1_* and θ*_1_* + π, or four balance mass points with masses of *M*/4 and positions of θ*_1_*, θ*_1_* + π/2, θ*_1_* + π, and θ*_1_* + 3π/2 are added to the imperfect resonator to eliminate the frequency split, as shown in Figure 9. Three elimination methods are compared in Figure 9. Figure 9a shows the distribution of balance mass points and imperfect mass points. Figure 9b shows the relationship between θ*_1_* and the frequency split. It is clear that the minimum frequency split position (θ*_1_*) is π/4 rad (*i.e.*, 45°), which draws the same conclusion as in Section 2. Figure 9c shows the relationship between *M* and the frequency split. The value of the frequency split is the smallest when the total mass of two or four balance points is 0.04 g, and when the mass of one balance point is 0.036 g. This result is a little different from our previous conclusion. Moreover, the method with four balance mass points turns out to be the best.

**Figure 10.**
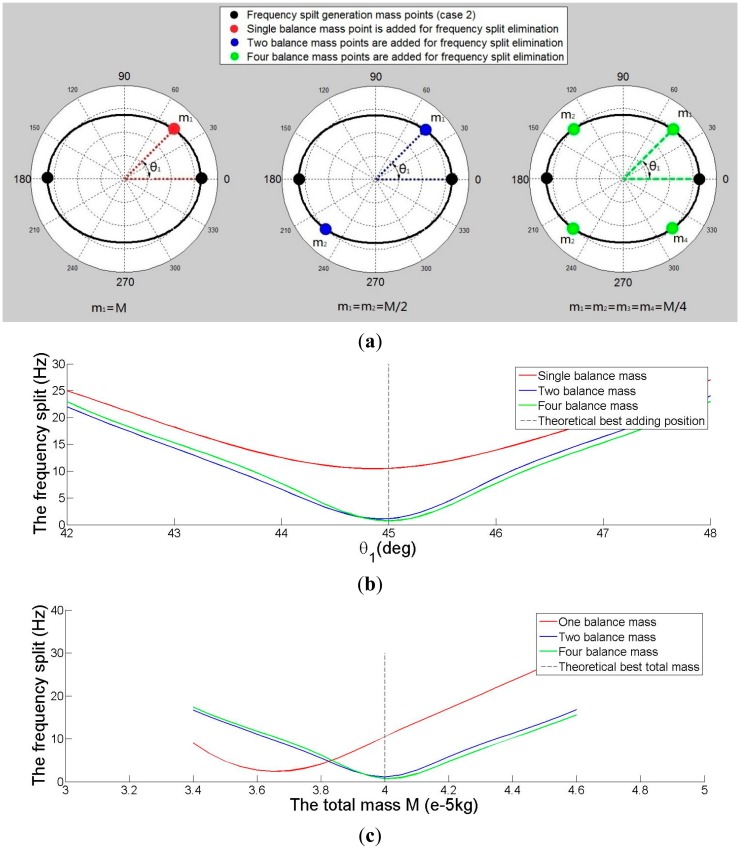
(**a**) The distribution of added mass points for eliminating frequency split Case 2; (**b**) The relationship between θ*_1_* and frequency split for eliminating frequency split Case 2 (*M* = 0.04 g); (**c**) The relationship between mass of balance points *M* and frequency split for eliminating frequency split Case 2 (θ*_1_* = π/4).

Then the method is applied to eliminate the frequency split of imperfect shell Case 2. Three elimination methods are also compared in Figure 10. Figure 10a shows the distribution of balance mass points and imperfect mass points. Figure 10b shows the relationship between θ*_1_* and the frequency split. When four balance mass points are added, the minimum frequency split occurs when position (θ*_1_*) is π/4 rad, but when one or two balance mass points are added, the minimum frequency split position is slightly less than π/4 rad. This result is a little different from the conclusion obtained above. Figure 10c shows the relationship between *M* and the frequency split. The value of the frequency split is the smallest when the mass of two or four balance points is 0.04 g, and when the mass of one balance point is 0.0365 g.

**Figure 11.**
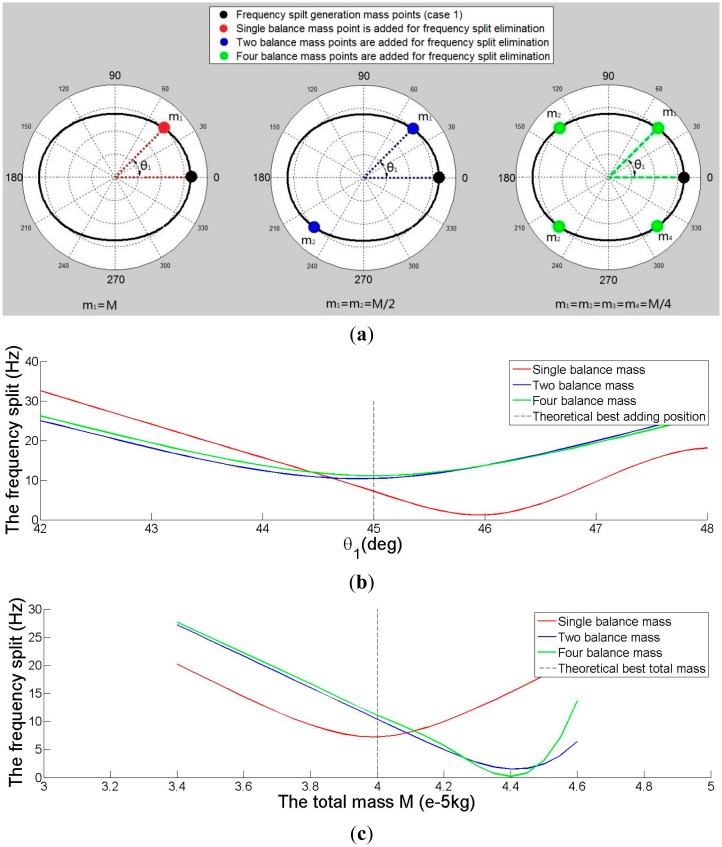
(**a**) The distribution of adding mass points for eliminating frequency split Case 1; (**b**) The relationship between θ*_1_* and frequency split for eliminating frequency split Case 1 (*M* = 0.04 g); (**c**) The relationship between mass of balance points *M* and frequency split for eliminating frequency split Case 1 (θ*_1_* = π/4).

Finally the method is applied to eliminate the frequency split of imperfect shell Case 1. Three elimination methods are compared in Figure 11. Figure 11a shows the distribution of added mass points. Figure 11b shows the relationship between θ*_1_* and the frequency split. When four balance mass points are added, the minimum frequency split occurs when position (θ*_1_*) is π/4 rad. The frequency split is minimum when one balance point is added and θ*_1_* is slightly over π/4 rad (about 46°). The minimum frequency split position (θ*_1_*) of two balance points is less than π/4 rad. Figure 11c shows the relationship between *M* and the frequency split. The minimum frequency split mass is 0.04 g when one balance point is added, or the mass is 0.044 g when two or four balance points are added.

### 3.3. Discussions

We can see that when the gap between the actual mode shape of an imperfect shell and the mode shape assumed by Equation (7) are not small enough, so the simulation results are a little different from the theoretical conclusion. It is clear that the imperfection does not only change shift angle ξ, but also changes the amplitude. The amplitude curve of an imperfect resonator is not a sine curve anymore. The minimum frequency split position and mass will change when the amplitude curve deviates from a sine curve. In the three frequency split cases, the amplitude curves of Cases 2 and 3 are the closest to a sine curve. In these two cases, the elimination method with four balance mass points works better and closely matches the theoretical result. This is because the points in the method with four balance mass points are more uniformly-distributed than in the method with one or two balance mass points, but for Case 1, the minimum frequency split position and mass all disagree with the theoretical analysis. This is due to the fact that the amplitude curve of Case 1 deviates from a sine curve, as shown in Figure 8. The less the deviation is, the closer the theoretical analysis and real situation will be, so when Case 1 occurs in a practical application, by adding mass at the position of the lower natural frequency mode's antinode with largest amplitude, we can adjust the amplitude of four antinodes to the same level. The amplitude curve will be then be closer to a sine curve and elimination methods with four balance mass points can be applied and the frequency split will be effectively eliminated.

## 4. Experiments and Verification

**Figure 12.**
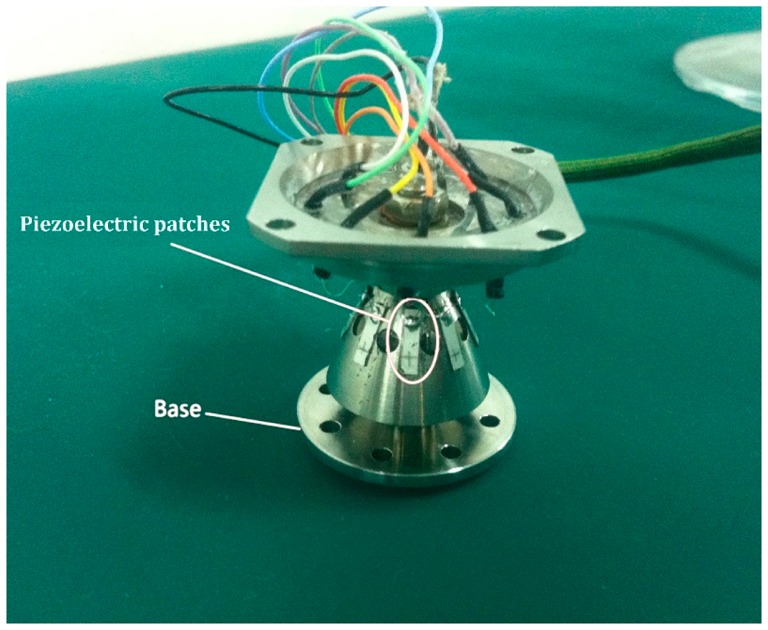
The real resonator and piezoelectric patches.

**Figure 13.**
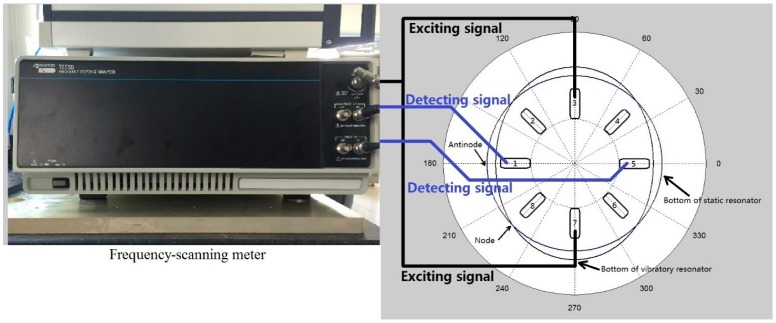
The instrument connection flow chart for natural frequency measurements.

The real resonator used in the experiment is shown in Figure 12. Piezoelectric patches are used to excite and detect the vibration of the resonator. The instrument connection flow chart for natural frequency measurement is shown in Figure 13. The piezoelectric patches are numbered. The piezoelectric patches chosen to excite electrodes have an angle of 180° with each other. For example, No. 3 and No. 7 are chosen as exciting electrodes in Figure 13. The piezoelectric patches which have an angle of 90°. with the exciting electrodes are chosen as detecting electrodes, for example, No. 1 and No. 5 are chosen in Figure 13. Natural frequency should be measured twice. If No. 1, No. 5, No. 3 and No. 7 patches are used as detecting and exciting electrodes in the first measurement, then No. 2, No. 6, No. 4 and No. 8 patches should be used as detecting and exciting electrodes in the second measurement.

**Figure 14.**
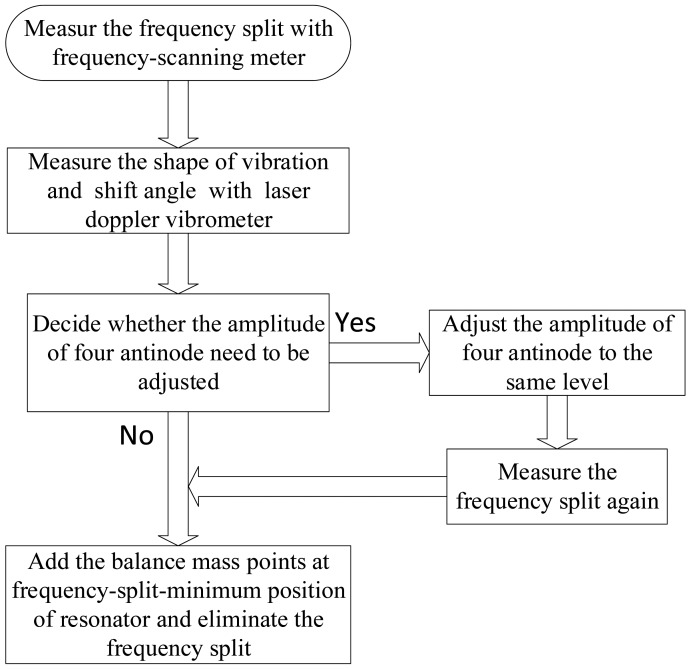
The flow chart of frequency split elimination.

**Figure 15.**
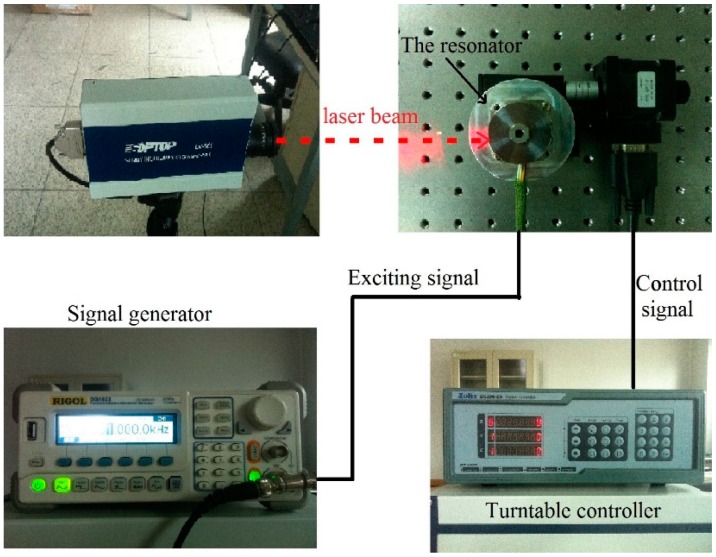
The instruments' connection flow chart for amplitude curve measurement.

Figure 14 shows the process of frequency split elimination. Firstly, the frequency split is measured by connecting these electrodes to a frequency-scanning meter. The output port of the frequency-scanning meter is connected to the exciting electrodes and the input port is connected to the detecting electrodes, as shown in Figure 13. Secondly, the amplitude curve is measured by a laser Doppler vibrometer. Figure 15 indicates the instruments' connection for amplitude curve measurement. The resonator is fixed on a turntable. The turntable is controlled by the controller which is shown at the bottom right of Figure 15. The signal generator generates the exciting signal. Then we should decide whether the amplitude of the four antinodes needs to be adjusted. This step is designed to solve the elimination problem when we are in practical situations similar to frequency split Case 1. We need to adjust the amplitude of the four antinodes to the same level. Finally, we add a balance mass at four frequency-split-eliminating positions with the same equivalent mass point mass.

**Figure 16.**
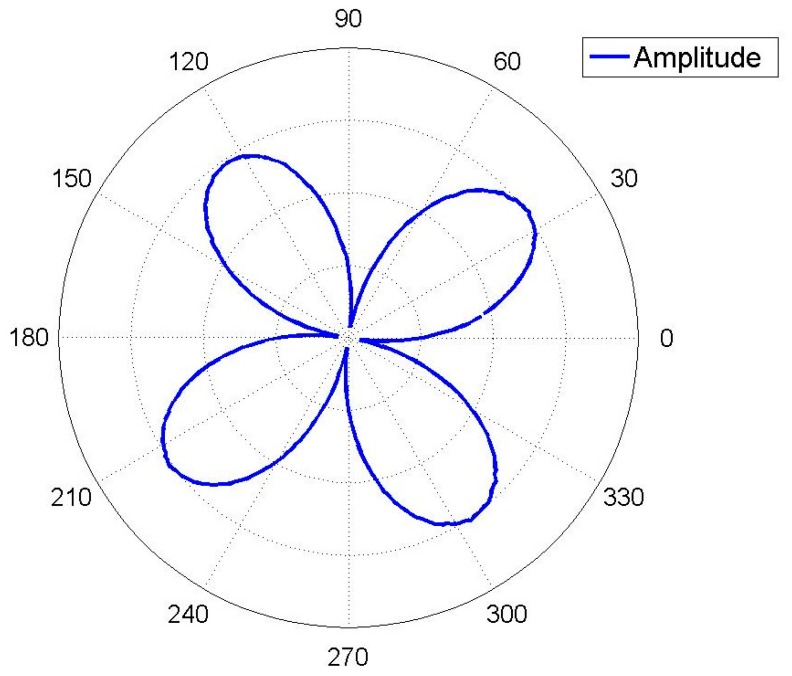
The amplitude curve measured by a laser Doppler vibrometer.

**Figure 17.**
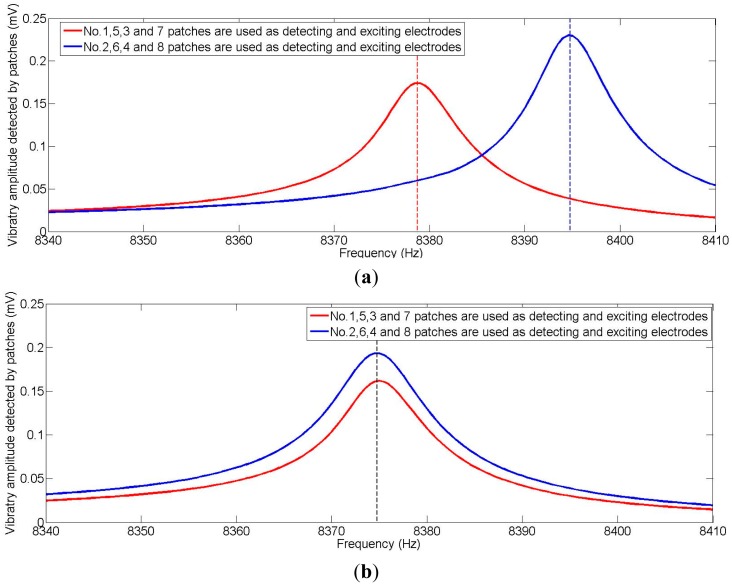
(**a**) Frequency spectrogram of the resonator before eliminating the frequency split; (**b**) Frequency spectrogram of the resonator after eliminating the frequency split.

Figure 16 shows the amplitude curve measured by a laser Doppler vibrometer at the lower natural frequency. Table 3 shows the node and antinode amplitude values. Surface flatness of the resonator, eccentricity of the bases and the bias of the laser beam will all lead to some measurement errors. After all, the amplitude curve is close to a sine curve, so according to the analysis above, the frequency-split-eliminating positions for adding mass are 81°, 171°, 261°, and 351°. The total mass of balance points can be calculated by Equation (15), though the procedure is complex. The equivalent imperfect mass is much smaller than the resonator, so the relationship between the mass of the equivalent imperfect mass points and the frequency split is almost linear. For the convenience of calculation, we can estimate the mass of balance points with experimental data. Figure 17 shows the frequency spectrogram of the resonator before processing and after processing. The frequency split is about 16 Hz (the frequency difference between two peaks) and four little blocks with the same mass are added at the bottom of the resonator with θ*_i_* at 81°, 171°, 261° and 351°. The frequency split drops below 0.5 Hz. The elimination method is thus proven very effective.

The mass and position of the balancing masses need to be controlled accurately when eliminating the frequency split. The Q-factor of the resonator may affect the procedure of the elimination method. Under the current Q-factor value, it is hard to measure the frequency split on the frequency spectrogram when the frequency split is below 1 Hz. A higher Q-factor is required when a smaller frequency split is needed.

## 5. Conclusions

This paper presents a way to analyze the frequency split problem of an axisymmetric multi-curved surface shell resonator. The concept of equivalent mass points is used to model the imperfections. The paper proposes an approach to eliminate the frequency split. The best balance position has an angle of 0.25π, 0.75π, 1.25π or 1.75π with respect to the antinode of the mode which has the lower natural frequency. The total mass of the balance mass point is equal to the equivalent imperfect mass point. FEM and experiments are used to verify the method's effectiveness. The approach can be used on other kinds of resonators with an axisymmetric thin shell structure because there is no requirement on the form of function U(φ), V(φ), W(φ) in Equation (3). The resonator can be used to detect the angular rate after being processed with this method.

## Figures and Tables

**Table 1.** The properties of Ni43CrTi.

**Name of Parameter**	**Value**
Density (kg·m^−3^)	8170
Poisson's ratio	0.3
Young modulus (GPa)	196.76

**Table 2.** (**a**) The impacts of latitude divisions on simulation results; (**b**) The impacts of longitude divisions on simulation results.

**(a)**				

**Number of Latitude Divisions (Longitude Divisions Is 60)**	**Perfect Shell (Unit: Hz)**		**Imperfect Shell (*m****_p_* **= 0.04 g) (Unit: Hz)**	
	
	**Higher Natural Frequency (ω***_nH_***)**	**Lower Natural Frequency (ω***_nL_***)**	**Higher Natural Frequency (ω***_nH_***)**	**Lower Natural Frequency (ω***_nL_***)**
120	6740.0	6740.1	6706.5	6581.4
180	6738.4	6738.3	6704.8	6579.8
360	6737.5	6737.4	6703.7	6578.9
720	6737.4	6737.2	6703.6	6578.9

**(b)**				

**Number of Longitude Divisions (Latitude Divisions Is 360)**	**Perfect Shell (Unit: Hz)**		**Imperfect Shell (*m****_p_* **= 0.04 g) (Unit: Hz)**	
	
	**Higher Natural Frequency (ω***_nH_***)**	**Lower Natural Frequency (ω***_nL_***)**	**Higher Natural Frequency (ω***_nH_***)**	**Lower Natural Frequency (ω***_nL_***)**

50	6737.6	6737.4	6703.8	6579.1
70	6737.6	6737.4	6703.8	6579.1
90	6737.6	6737.4	6703.8	6579.1
110	6737.6	6737.4	6703.8	6579.0

**Table 3.** The amplitude measured by a laser Doppler vibrometer.

**Antinode Position (°)**	**Amplitude (nm)**	**Node Position (deg)**	**Amplitude (nm)**
36	536.1	81	16.1
126	536.7	171	21.8
216	531.9	261	14.2
306	533.3	351	17.1
